# Removal of Doxycycline from Water using *Dalbergia sissoo* Waste Biomass Based Activated Carbon and Magnetic Oxide/Activated Bioinorganic Nanocomposite in Batch Adsorption and Adsorption/Membrane Hybrid Processes

**DOI:** 10.1155/2022/2694487

**Published:** 2022-03-17

**Authors:** Muhammad Zahoor, Muhammad Wahab, Syed Muhammad Salman, Aamir Sohail, Essam A. Ali, Riaz Ullah

**Affiliations:** ^1^Department of Biochemistry, University of Malakand, Chakdara Dir Lower, Khyber Pakhtunkhwa, Pakistan; ^2^Department of Chemistry, Islamia College University, Peshawar, Khyber Pakhtunkhwa 25000, Pakistan; ^3^MSC Construction Project Management, University of Bolton, Bolton, UK; ^4^Department of Pharmaceutical Chemistry, College of Pharmacy, King Saud University, Riyadh, Saudi Arabia; ^5^Department of Pharmacognosy, College of Pharmacy, King Saud University, Riyadh, Saudi Arabia

## Abstract

The carbonaceous adsorbents, an activated carbon (AC) and a bioinorganic nanocomposite (MAC), were prepared using *Dalbergia sissoo* sawdust as waste biomass, in this study. Both the adsorbents were characterized by FTIR, EDX, SEM, XRD, TG/DTA, surface area, and a pore size analyzer. The adsorbents were used for the removal of an antibiotic, doxycycline (DC) antibiotic, from wastewater in order to minimize a load of antibiotics in industrial effluents and consequently the drug resistance problem. Initially, the effectiveness of adsorbent was confirmed using batch adsorption experiments where isothermal models like Langmuir, Freundlich Temkin, Jovanovic, and Harkins–Jura were utilized to govern the maximum adsorption capacity of AC and MAC while pseudo-first- and second-order kinetic models were used to estimate the values of different kinetic parameters. Langmuir model best accommodated the equilibrium data whereas the pseudo-second-order kinetic model finest trimmed the kinetics data. The effect of pH on adsorption was also evaluated where maximum removal was observed between pH 5 and 7 by both adsorbents. The effect of temperature on adsorption was evaluated where the entropy change (Δ*S*^0^) comes out to have a numerically positive value whereas Gibbs free energy change (Δ*G*^0^) and enthalpy change (Δ*H*^0^) were negative indicating the spontaneous nature and feasibility of the procedure. The robust technology of membrane separation is rapidly replacing the conventional technologies but at the same time suffers from the problem of membrane fouling. As pretreatment, the AC and MAC were used in hybrid with ultrafiltration (UF), nanofiltration (NF), and reverse osmosis (RO) membranes whereas permeate fluxes and percent retention of DC were compared for naked membrane operations and AC/membrane and MAC/membrane process. The permeate fluxes for MAC/membrane processes were greater as compared to AC/membrane and naked membrane processes showing the effectiveness of the bioinorganic composite as foul control and consequently recovery of DC from effluents. The percent retention of the UF membrane was lower as compared to NF and RO membranes. Improvement in percent retention for UF/AC, UF/MAC, NF/AC, NF/MAC, RO/AC, and RO/MAC was observed. The bioinorganic composite MAC contains a magnetic iron oxide which was effectively removed from slurry after use through the magnetic process and that was the main reason for high permeate fluxes in MAC/membrane operations.

## 1. Introduction

Pharmaceutical compounds are continuously added to the environment from pharmaceutical industries, hospital wastewater, veterinary health care center, and domestic wastewaters [1] which have negative impacts on aquatic animals and can enhance the problem of drug resistance as the microbes living in such waters with times adapt themselves according to antibiotics containing changed environment. Doxycycline (DC) is an antibiotic that targets both Gram-positive and Gram-negative bacteria that is used for the curing of pelvic inflammatory malady, syphilis, and chronic prostatitis [2]. Inside human and animal bodies, DC is not completely metabolized (20 to 50% is metabolized) and the remaining is excreted unaltered into the environment. That is why the remains of DC have been repeatedly noticed in surface water, groundwater, and even in soil [3]. It has been confirmed that the contact of even a low level of DC kills aquatic photosynthetic microbes, local microbial populations, and the propagation of antibiotic-resistant genes among microbes [4–6]. Numerous methods have been secondhand for antibiotics and heavy metals removal from the aquatic environment such as advanced oxidation, ozone oxidation, ions exchange, precipitation, adsorption, and membrane hybrid methods [7, 8]. Among these methods, the adsorption strategy is highly efficient, easy to operate, and cheap and has no risk of toxic byproducts [9]. Therefore, it can be used as one of the most active approaches for antibiotic and other pollutants' removal from the aquatic environment. Various adsorbents such as clay-biochar, modified cellulose nanofibers, silica gel, zeolite, and rice husk have been used for antibiotic removal from the aquatic environment [10, 11]. Activated carbon has a high surface area and is, therefore, the most efficient adsorbent. However, due to lighter weights, it has longer settling times in reactors and one would have to wait for a few days to separate them from slurry after use in industry. To remove this inconsistency, many researchers have prepared bioinorganic composites containing magnetic oxides with an idea that such composites have a comparable surface area as that of activated carbon and due to their presence oxide can simply be separated from slurry after usage through the application of magnet [12].

The above-mentioned methods are conventional methods which are not rapid and take time. On the other hand, membrane technologies are the rapid approaches that can be used in line with the rapid reclamation of industrial effluents [12]. Different membranes such as ultrafiltration (UF), nanofiltration (NF), and reverse osmosis (RO) have been employed for the removal of different pollutants. However, membranes are porous structures whose pores may be blocked by pollutants having a size larger than membrane pores which may be deposited over there due to concentration polarization and adsorption. This phenomenon is known as membrane fouling. To ensure the effective use of membranes in industries, activated carbon (AC) pretreatments were suggested by researchers. Initially, it was assumed that if activated carbon enters into the membrane system, this would not affect the membrane efficiencies but later on a negative effect of activated carbon was observed on permeate flux. To solve this issue, Zahoor et al. utilized magnetic oxide/AC composite as pretreatment where improved permeate fluxes were observed. Because of magnetic character, the entrance of such adsorbent into membrane structure was stopped through the usage of the magnet [13–15]. In the present research study, two carbonaceous adsorbents AC and a bioinorganic composite MAC were set from sawdust of *Dalbergia sissoo* and were applied as pretreatment for effective removal of DC from wastewater to regulate membrane fouling instigated by DC in industries.

## 2. Experimental Setup

**Figure 1:**
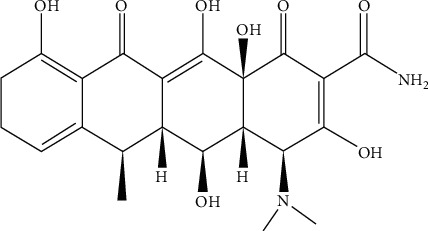
Chemical structure of doxycycline.

DC having formula C_22_H_24_N_2_O_8_ (Figure 1; Mol. Weight = 444.44 g/mol; *λ*_max_ = 275 nm) is a synthetic derivative of tetracycline which was introduced by Pfizer in the year 1970 [16] generously donated by Medi-craft industry, Hayat Abad, KP, Pakistan, and utilized without any more refinement. It was developed as a second-generation antibiotic to lower the toxicity of its parental first-generation tetracycline through substitution of 5*β*-H with OH group and 6*α*-OH by H.

### 2.1. Preparation of Adsorbents

For the preparation of *Dalbergia sissoo* sawdust-based AC and MAC, the raw biomass was brought from a local mill in Bajaur Agency, Pakistan, dried in an oven, and further ground into fine powders. While preparing AC, no pretreatment was applied and ignited in a specially designed chamber in nitrogen atmosphere whereas in MAC preparation a pretreatment of the dried powders was applied, consisting of immersing the powders in a 400 ml mixture of iron sulfate and iron chloride (200 ml each) which were then percolated with 100 ml NaOH in dropwise from a burette at 70°C. The pH of the mixture was brought to neutral through repeated washing with distilled water. After drying in the oven, the resulting mass was ignited as per the procedure described above. The black-colored adsorbents (AC and MAC) obtained were then kept in airtight bottles till their use in the targeted experiments.

### 2.2. Characterization of Adsorbents

The prepared adsorbents such as AC and MAC were analyzed for elemental composition using the EDS X-sight instrument (JEOL USA JSM—5910). The functional groups present in AC and MAC were investigated by using the Perkin Elmer FTIR instrument (ranging from 400–4000 cm^−1^). The morphology of AC and MAC was investigated using SEM, model JSM 59 JEOL, Japan. XRD model JDX-3532 JEOL, Japan, was used to confirm magnetite/maghemite in MAC. The thermogravimetric and differential thermal analysis was performed using a thermogravimetric analyzer (Pyres Diamond Series TG Perkin Elmer, USA), up to 800°C. The particle sizes of the adsorbents, pores' distribution, and consequently their surface areas were determined using an analyzer of type NOVA2200e, Quantachrome, USA.

### 2.3. Batch Adsorption Experiment

Batch adsorption was initially applied to estimate adsorption isothermal and kinetics parameters whereas the same approach was used for estimating thermodynamics parameters and optimizing adsorbent dosage, etc. In almost all the mentioned experiments, 50 ml fixed concentration of DC solution was contacted with a fixed amount of both adsorbents in 250 flasks with the exception of isothermal studies where different concentrations of DC (20–300 mg/L) in 50 ml volume were contacted with a fixed amount of adsorbent. In optimizing pH, the mixture pH was adjusted per requirement (2–12) with 0.1 M HCl/NaOH; while optimizing the adsorbent dosage, the amount of the adsorbent was changed from 0.01 to 0.15 g. In case kinetics experiments, samples were withdrawn at different time intervals (0–240 min). The temperature effect (estimating thermodynamics parameters) was studied within a range of 293–333 K. The shaker speed in all experiments was 170 rpm. After completion of each experiment, the remaining concentration of DC in solution was determined through a UV/Vis spectrophotometer (T-60, PG, UK) at 275 nm. The amount of selected antibiotic adsorbed and, consequently, its percent removal were recorded through the following relations [17, 18]:

(1) $$\begin{array}{c} q_{e}=\left(C_{i}-C_{f}\right)\times \frac{V}{m}, \end{array}$$

(2) $$\begin{array}{c} \%\;R=\frac{\left(C_{i}-C_{f}\right)}{C_{i}}\times 100. \end{array}$$

In equations (1) and (2), *C*_*i*_ stands for initial concentration, *C*_*f*_ for final equilibrium concentration of antibiotic used, *V* for solution volume (*L*), and *m* for mass (g) of AC and MAC.

For the validation of isothermal data, Langmuir, Freundlich, Temkin, Jovanovic, and Harkins–Jura have been used whereas pseudo-first-, second-order, power function, Natarajan–Khalaf, and intraparticle diffusion have been applied to get the best fit of the kinetics data.

### 2.4. Robust Removal of DC with Membranes with and without Pretreatment of Adsorbents

Initially, all the three membranes (UF, NF, and RO) mounted on a steel stand were rinsed with distilled water for 30 min each to note the intrinsic resistance of membranes to water; then, 100 mg/L DC solution was passed from each membrane separately to note its %Retention and consequently their fouling effect in terms of reduction in permeate fluxes, using following formulae [19]:

(3) $$\begin{array}{c} \%\;R=\left(1-\frac{C_{p}}{C_{b}}\right)\times 100, \end{array}$$

(4) $$\begin{array}{c} J=\frac{1}{A}\times \frac{\text{d}v}{\text{d}t}. \end{array}$$

In these equations, *C*_*p*_ and *C*_*b*_ (mg/L) represent concentrations of DC in permeate and feed, i.e., bulk, respectively, *J* represents permeate flux, A is the area of the given membrane in m^2^, *V* is the volume of permeate in L, and *t* is time.

As pretreatment, AC and MAC were mixed in feed bulk in specific proportion as optimized in batch experiments, in a continuously stirred reactor, and after equilibrium, time attainment was channelized into membranes and the above-mentioned parameters in equations (3) and (4) were calculated. MAC, due to its magnetic character, after equilibration was stopped from being entered into the membrane module. The characteristic properties of membranes used are given in Table S1 whereas a sketch of the pilot plant is given in Figure S1.

## 3. Result and Discussion

### 3.1. Characterization of Adsorbents

**Figure 2:**
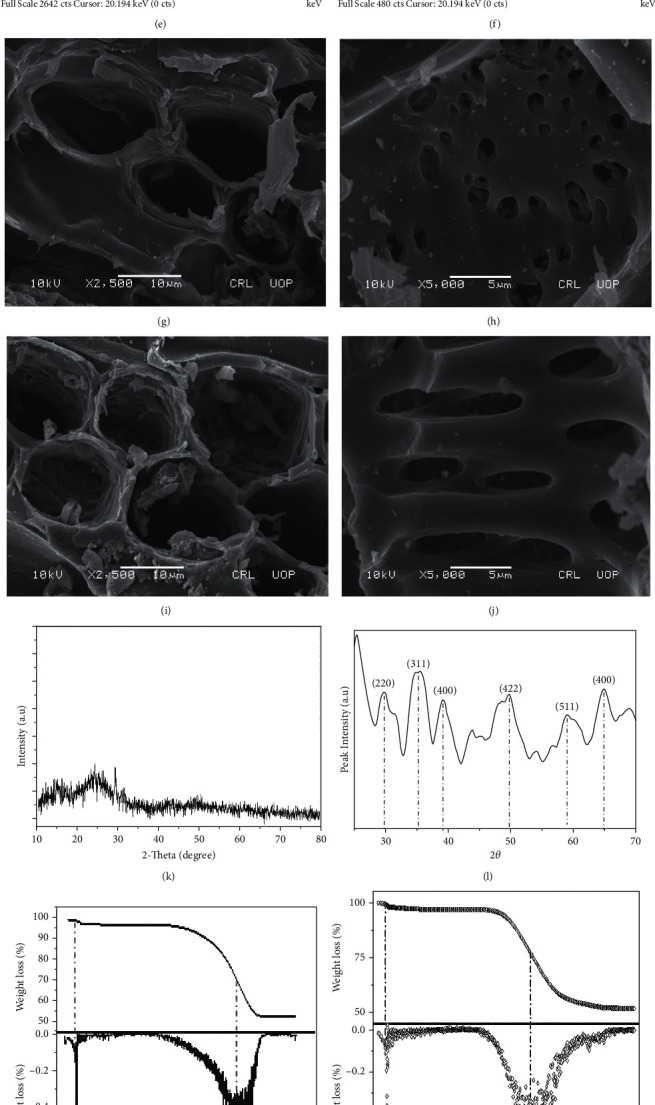
Instrumental characterization of AC and MAC. (a) FTIR spectrum of AC. (b) FTIR spectrum of MAC. (c) FTIR spectrum of DC adsorbed by AC. (d) FTIR spectrum of DC adsorbed MAC. (e) EDX spectrum of AC. (f) EDX spectrum of MAC. (g, h) SEM images of AC. (i, j) SEM images of MAC. (k) XRD graph for AC. (l) XRD graph for MAC. (m) TG/DTA graph for AC. (n) TG/DTA graph for MAC. (o) BET surface area for AC. (p) BJH pore size distribution for AC. (q) BET surface area for MAC. (r) BJH pore size distribution for MAC.

The prepared bioinorganic composite MAC and parental material-based AC were characterized by various instrumental techniques to compare the morphological and structural differences between the two. FTIR spectra which are normally used to determine surface functional groups of adsorbents are shown in Figures 2(a) and 2(b). The C≡C stretching is evident from the peak appearing between 2250 and 2300 cm^−1^ whereas the O-H group presence was confirmed from the peak appearing between 3200 and 3400 cm^−1^. The isothiocyanate presence can be confirmed from a peak at 2150 cm^−1^. The methylene group stretching was observed at 2900 cm^−1^. The methoxy/methyl ether stretching appeared at 1850 cm^−1^. The presence of the aldehyde group is evident from the peak at 1700–1800 cm^−1^ whereas a peak between 1580 and 1675 cm^−1^ shows the C=C stretching in AC. The presence of the organic sulfate group was assumed from peaks at 1370 and 1420 cm^−1^. The aromatic stretching appeared at 1510–1450 cm^−1^. The most important peak at 700 cm^−1^ in MAC showed the Fe-O bond confirming the deposition of iron oxide in the composite structure. The interaction of selected antibiotic DC after adsorption has shifted the peak positions for both the adsorbents as shown in Figures 2(c) and 2(d) indicated the positive interactions between the adsorbent and adsorbate. In our previous publications, although such shifting was observed, here, the interaction is more favorable that has resulted in high adsorption capacity and consequently better effects on foul control in the membrane processes [20–22]. The interactions between adsorbent functional groups and those of adsorbate play an important role in the reclamation of a given pollutant from an aqueous environment. When there is shifting of many functional group peaks after adsorption of a given pollutant, the interactions are considered more favorable in comparison to a system where the shifting is not drastic. In the case of our previous study, the shifting was not so significant as compared to those observed in the present study.

As our study was aimed to prepare a magnetic composite, therefore, elemental analysis was performed to confirm the presence of iron in MAC in particular and also to compare the elemental composition of it with parental biomass-based AC (Figures 2(e) and 2(f)). Carbon and oxygen are the main components in AC whereas small amounts of calcium, magnesium, and phosphorus were evident from broad and small peaks, respectively. The peak of iron in MAC EDX spectra confirmed the deposition of iron oxide on the surface of AC that has resulted in lower carbon contents MAC as compared to AC. MAC elemental spectra also indicated elements like chlorine and oxygen along with a low percentage of calcium.

To visualize the surface morphology, SEM photographs were taken for both AC and MAC (Figures 2(g)–2(j)). SEM pictograms of AC show porous structure with crooks and flattened edges distributed in an irregular fashion. A similar porous structure of MAC is evident from the given figures whereas additional dots scattered on the surface of MAC represent the cubic crystalline Fe_3_O_4_.

All iron oxides are not attracted by a magnet. Only magnetite and maghemite are attracted by the magnet. To confirm the magnetic character of the MAC, XRD analyses were performed. AC being an amorphous substance could not give any peak in XRD spectra whereas in MAC cubic iron oxide crystal was there in its structure resulting in a number of peaks in XRD spectra (Figures 2(k) and 2(l)). The peaks at 2*θ*: 29, 36, 38, and 50 in MAC spectra correspond to refractive indices: 220, 311,400, and 422, respectively, confirming that the magnetite is present in the composite structure.

The thermal stability of AC was evaluated through TG/DTA analysis as shown in Figure 2(m). In the initially taken mass of 5.441 mg, there was a 60% loss up to 60°C which is actually the loss of absorbed water followed by another 50.35% second mass loss from 60 to 450°C which most probably represents the decomposition of cellulose and hemicellulose in the biomass. A third mass loss of 33.99% as presented in the last portion of the spectra is due to the conversion of biomass into carbon and ash. An initial mass was 5.663 mg of MAC when was subjected to TG/DTA analysis; a 3% loss was observed from 0 to 60°C as shown in Figure 2(n), which is actually evaporation of water followed by a 7% loss observed from 60 to 318°C as a result of thermal degradation of cellulose material in composite. The third mass loss (58%) was observed above 318°C where carbonaceous residue formation has taken place along with probability of Fe_3_O_4_ conversion to FeO.

The BET and BJH plots of AC (49.53 and 21.76 m^2^/g) and MAC (18.63 and 41.97 m^2^/g) are shown in Figures 2(o)–2(r), respectively. The high surface area values of AC in comparison to MAC are the result of the impregnation of Fe_3_O_4_ on the MAC surface that has resulted in lower carbon contents in an equivalent mass of MAC. The BJH pore distribution showed that the pore volume and pore radius of AC, respectively, were equal to 0.019 cc/g and 16.11 A^º^ whereas for MAC they were 0.016 cc/g and 14.85 Aº, respectively [23–25].

### 3.2. Isothermal Studies

Such types of studies are performed to get an insight into adsorption processes and to estimate parameters like adsorption capacity monolayer or multilayer formation by given contaminants, etc. which are decided from different models as mentioned in the experimental section.

#### 3.2.1. Freundlich Isotherm

This model is actually based on an empirical equation that most favorably describes the heterogenous adsorption systems and assumes that the active adsorption sites are distributed exponentially for a given adsorbent. The equation is applied in a number of different forms but the most used one that is the linear form has been used in this study [26]:

(5) $$\begin{array}{c} \ln\;q_{e}=\ln\;aK_{F}+\frac{1}{n}\ln\;C_{e}. \end{array}$$

**Figure 3:**
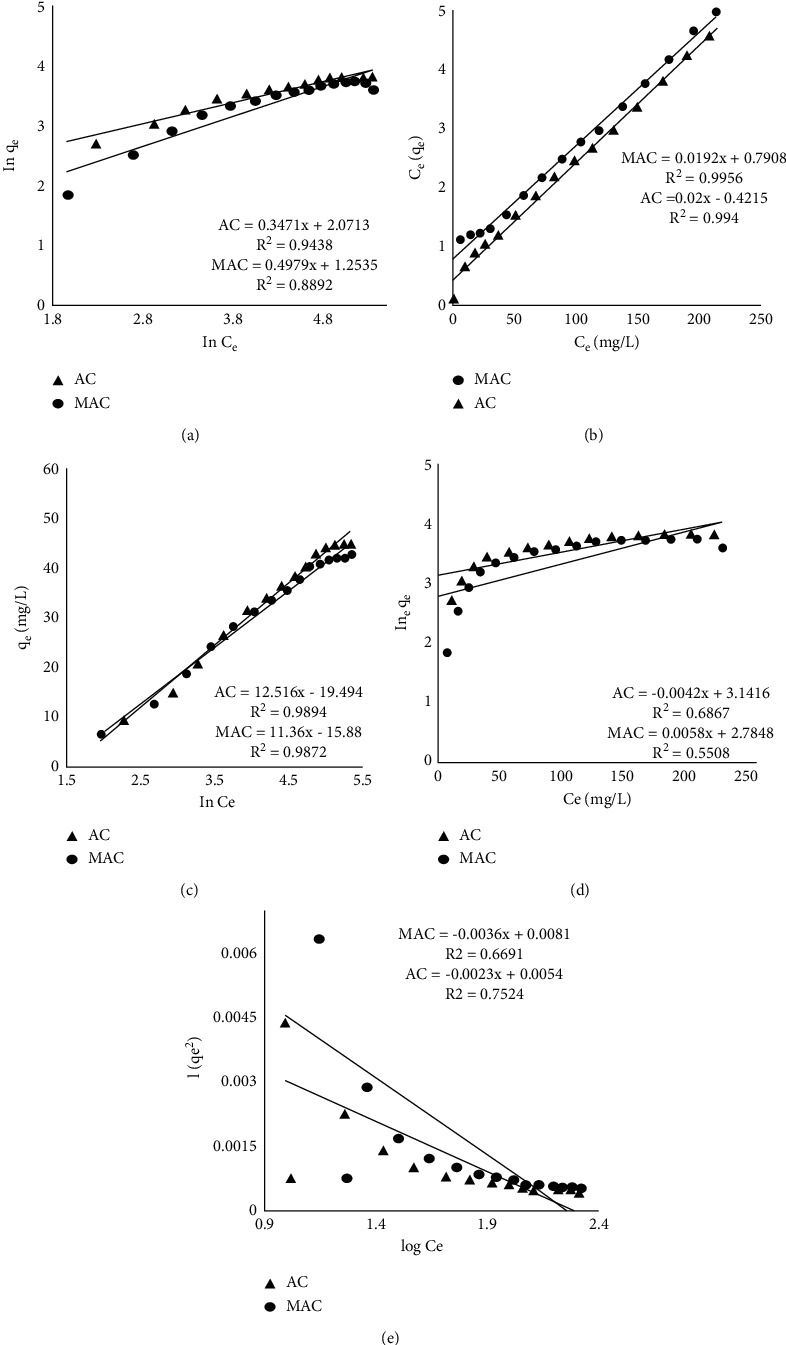
Adsorption isotherms: (a) Langmuir, (b) Freundlich, (c) Temkin, (d) Jovanovic, and (e) Harkins–Jura.

The abbreviation in this equation represents the following: *q*_*e*_ (mg/g) is the amount of DC adsorbed by adsorbents, *K*_*F*_ (L/mg) is the capacity of adsorption of AC and MAC, and 1/*n* is the adsorption intensity. The latter two parameters are calculated from the slope and intercept correspondingly from the graph given in Figure 3(a) whereas Table 1 summarizes their estimated values.

#### 3.2.2. Langmuir Isotherm

This isotherm more likely describes the monolayer adsorption processes where it is presumed that the adsorbent has finite surface-active sites of uniform dimensions, and after getting engaged with given pollutant molecules, no interactions from laterally adsorbed molecules are expected [27]. Equation (6) represents the linear form of this model:

(6) $$\begin{array}{c} \frac{C_{e}}{q_{e}}=\frac{1}{K_{L}q_{m}}+\frac{C_{e}}{q_{m}}. \end{array}$$

The equation parameters represent the following: *q*_*e*_ (mg/g) is the equilibrium adsorbed amount of the antibiotic, *C*_*e*_ (mg/L) is the remaining concentration after getting equilibrium, *q*_max_ is the maximum removal capability of adsorbent in mg/g, whereas *K*_*L*_ is the Langmuir constant. The last-mentioned two parameters are estimated (Table 1) from the slope and intercept, respectively, from the given graph in Figure 3(b).

#### 3.2.3. Temkin Isotherm

This model (equation (7)) specifically describes the variation of the heat of adsorption which is considered to be decreased linearly with the progression of adsorption [28].

(7) $$\begin{array}{c} q_{e}=\beta \;\ln\;\alpha +\beta \;\ln\;C_{e}. \end{array}$$

The factor *β* is a complex number that I mathematically is equal to RT/b whereas in this relation *R* is equal to 8.314 J/mol.K numerically and is the universal gas constant, *T* is the temperature in Kelvin while *b* represents the heat associated with the adsorption process.  Figure 3(c) represents the graphical form of this equation where the slope is *β* and the intercept is equal to *β*ln*α* (Table 1).

#### 3.2.4. Jovanovic Adsorption Isotherm

This isotherm is actually a modified form Langmuir model as presented in equation (8) which in contrast considers some mechanical association between the sorbent and sorbate [29].

(8) $$\begin{array}{c} \ln\;q_{e}=\ln\;q_{\max}-K_{j}C_{e}. \end{array}$$

With the exception of *K*_*J*_, all ligands are defined above, whose value can be determined from the slope of Figure 3(d) along with *q*_max_ from its intercept (Table 1).

#### 3.2.5. Harkins–Jura Isotherm

This isotherm is heterogenous systems where multiple-layer formation possibility is high. Equation (9) represent its linear form [30].

(9) $$\begin{array}{c} \frac{1}{q_{e^{2}}}=\frac{B_{H}}{A_{H}}-\frac{1}{A_{H}}\log\;C_{e}. \end{array}$$

The given parameters *q*_*e*_ and *C*_*e*_ are already defined before whereas the remaining *B*_*H*_ and *A*_*H*_ constants are gotten from the slope and intercept of the plot shown in Figure 3(e) (estimated values are given in Table 1).

### 3.3. Adsorption Kinetic Study

Industrially, the kinetics of adsorption processes are very important as a prompt recovery of pollutants saves the time and economy of the owners. The kinetics have been evaluated for two different concentrations (50 and 100 mg/L).

**Figure 4:**
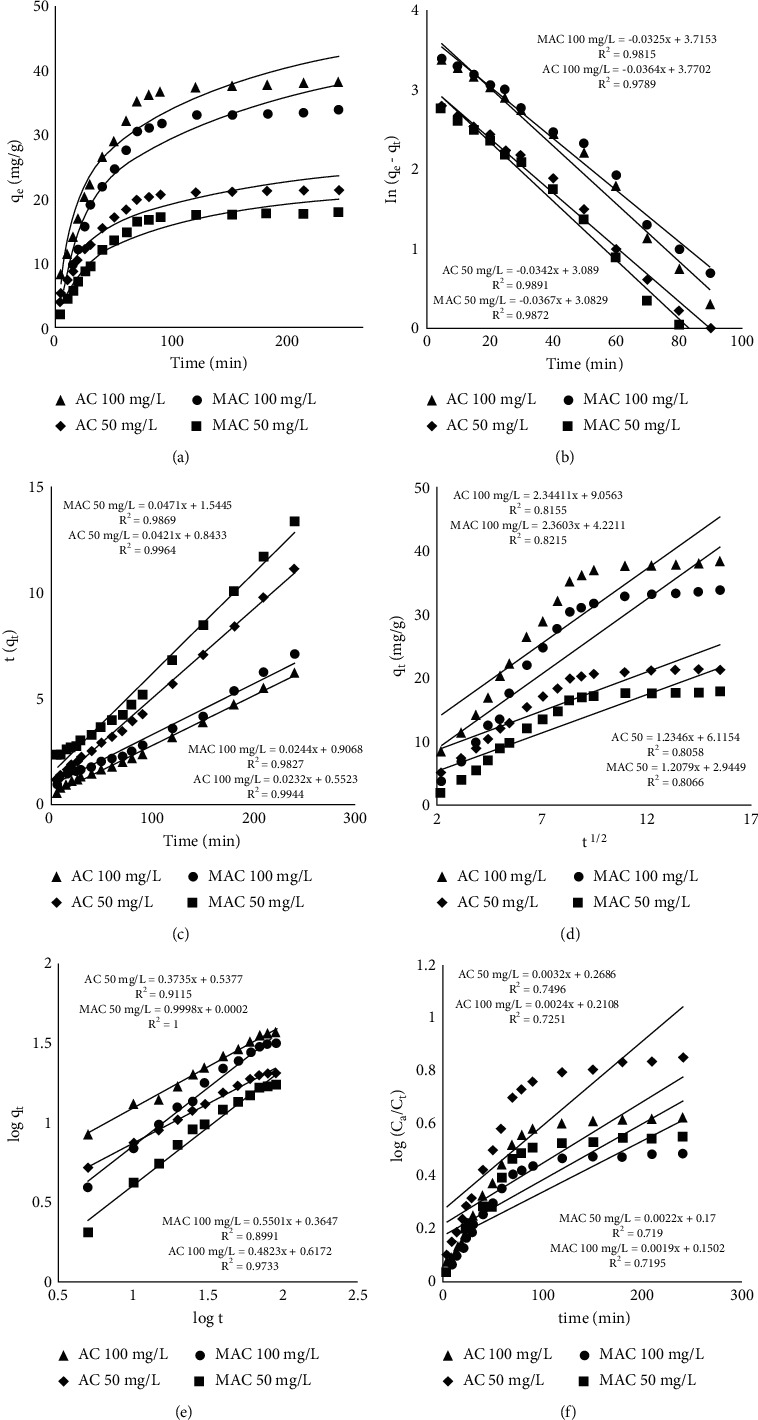
Adsorption kinetics. (a) Effect of time on sorption. (b) Pseudo-first-order kinetic model graph. (c) Pseudo-second-order kinetic model graph. (d) Intraparticle diffusion model graph. (e) Power function kinetic model graph. (f) Natarajan and Khalaf kinetic model graph.

Figure 4(a) is about equilibrium time which has been reached in 2 h for both adsorbents; however, most of the process has been completed in initial 30 min due to high availability adsorption sites which then gradually decrease till reaching an equilibrium where then sorption and desorption rates were equal.

The data from the above experiment was fitted into the following equation which is the linear form of the pseudo-first-order model [31].

(10) $$\begin{array}{c} \ln\left(q_{e}-\;q_{t}\right)=\ln\;q_{e\;}-K_{1}t. \end{array}$$

*K*_*1*_ (1/min) is the first-order constant, *q*_*e*_ is already defined above, and *q*_*t*_ is the amount of DC adsorbed at any time *t*, where the values of the first two parameters given in Table 2 have been calculated from the slope and intercept of the plot shown in Figure 4(b).

The same data was also inserted in pseudo-first-order equation (equation (11)) that is mostly applied to explain adsorption processes where chemical interactions between sorbate and sorbent are most likely involved [32]:

(11) $$\begin{array}{c} \frac{t}{q_{t}}=\frac{1}{K_{2}q_{e^{2}}}+\;\frac{t}{q_{e}}. \end{array}$$

With the exception of *K*_*2*_ which is a second-order equation constant and was calculated from intercept (Table 2) of the plot given in Figure 4(c), all other parameters in equation (11) are defined above.

The kinetic constants along with other parameters given in Table 2 show that regression coefficient values of the 2^nd^-order model are quite near to 1 showing the involvement of chemical interaction in the sorption of DC by both adsorbents.

To understand the underlying adsorption mechanism, the intraparticle diffusion model (equation (12)) was employed. Usually, adsorption is a two-step process where in the first step the sorbate transfers from solution to surface pore while in the second which is a slow step the sorbate penetrates into pores through diffusion [33].

(12) $$\begin{array}{c} q_{t}=K_{\text{diff}}t^{1/2}+\;C. \end{array}$$

In this equation, *K*_diff_ (mg/g.min^1/2^, Table 2) can be estimated from the slope (Figure 4(d)) and *C* is constant providing information about boundary layer thickness and can be estimated from the intercept of the given figure whereas *q*_*t*_ is already defined in other models.

Another model called the power function model was also applied that is mathematically presented as equation (13) that is the Freundlich model modified from [34].

(13) $$\begin{array}{c} \log\;q_{t}=\log\;a+b\;\log\;t. \end{array}$$

Here “*a*” represents the initial rate that can be estimated from the intercept of Figure 4(e) whereas “*b*” represents the reaction rate, as given in Table 2, and was estimated from the slope of the mentioned figure.

Natarajan and Khalaf's model was also applied as given below in its linear form [35].

(14) $$\begin{array}{c} \log\left(\frac{C_{o}}{C_{t}}\right)=\;\frac{K_{N}}{2.303}t. \end{array}$$

*C*_*o*_ (mg/L) in this equation is the initial concentration before starting the experiment, *C*_*t*_ (mg/L) is the concentration at time *t*, and *K*_*N*_ is the constant estimated from the slope of log (*C*_*o*_/*C*_*t*_) against *t* plot (Figure 4(f)).

### 3.4. Effect of pH on Sorption

**Figure 5:**
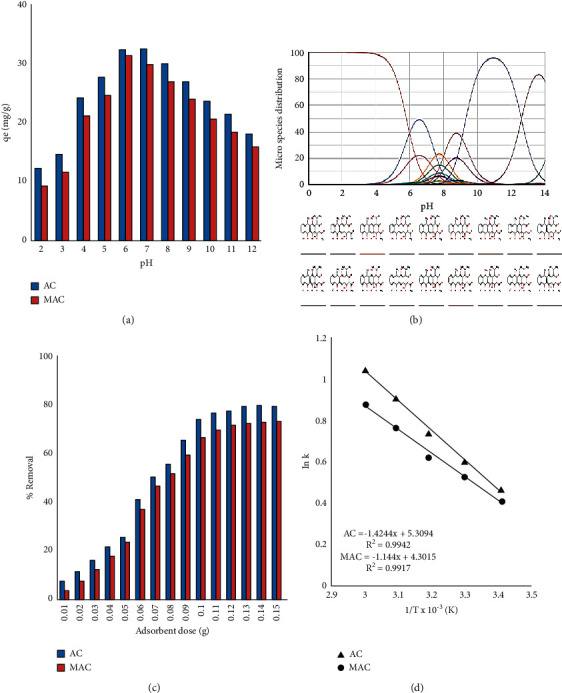
(a) Effect of pH. (b) speciation graph for DC. (c) effect of adsorbents dosage. (d) Van't Hoff's graph.

**Figure 6:**
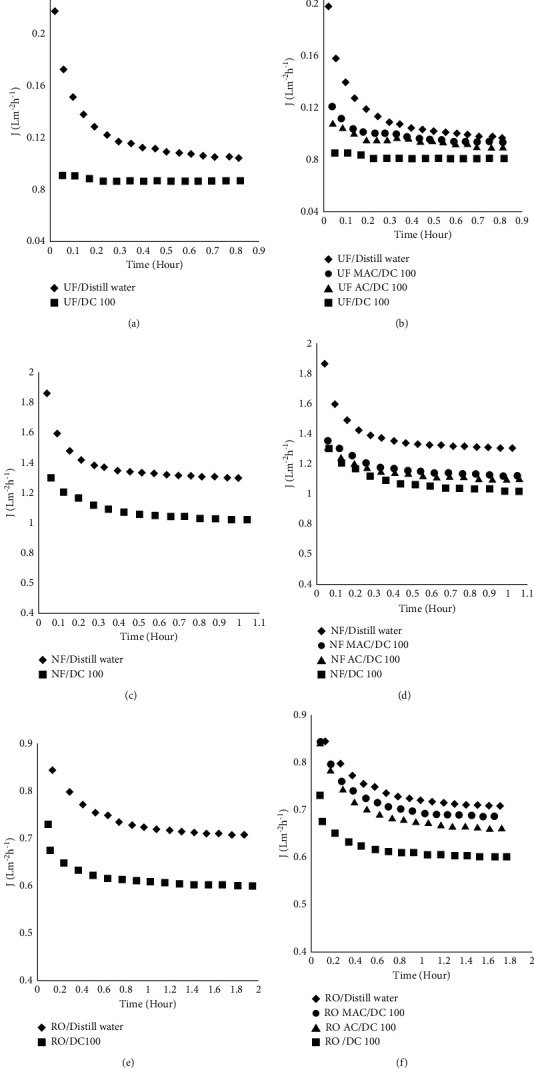
Effect of DC on permeates flux and improvement brought about by AC and MAC. (a) UF membrane alone. (b) AC/UF and MAC/UF. (c) NF membrane alone. (d) AC/NF and MAC/NF. (e) RO membrane alone. (f) AC/RO and MAC/RO.

Figure 5(a) shows that there is variation in the rate of DC adsorption when pH was changed from 2 to 12. Almost similar trends in pH dependency were observed for both adsorbents. In the range of pH 5–7, maximum adsorption was recorded whereas at lower pH values the sorbate and sorbent both were positively charged that had led to a lower rate of adsorptions, i.e., due to negative interactions between the two as predicted from FTIR spectra and DC speciation graph given in Figure 6(b). Similarly, negative charge dominancy at higher pH is evident from Figure 5(b) [36]; that is why optimum adsorption has been taken place near neutral pH.

### 3.5. Effect of Adsorption Dosage

The effect of sorbent measure is given in Figure 5(c) showing that maximum sorption has occurred with 0.1 g amount that was then used in almost all subsequent experiments. The initial increase in sorption rate with the rise in adsorbent dose may be considered due to surge in active sites whereas above doses from optimum did not bring any further increase due to scarcity of DC molecules in solution as fixed concentration solutions were used [37].

### 3.6. Thermodynamics of the Process

For given processes/reactions, the thermodynamic considerations are important as they give the most important information like spontaneity and feasibility about the process, and Van't Hoff relation (equation (15)) is used for reaching such important information mentioned in terms of ∆H° (change in enthalpy), ∆S° (change in entropy), and ∆G° (change in Gibbs free energy) [38].

(15) $$\begin{array}{c} \ln\;K=\;\frac{\Delta S^{o}}{R}-\frac{\Delta H^{o}}{RT}. \end{array}$$

*K* in this relation is partition constant that can be estimated as *K* = (*q*_*e*_/*C*_*e*_), *R* is the general gas constant (8.314 KJ/mol), and *T* represents temperature in Kelvin. ∆*H*^*o*^ and ∆*S*^*o*^ were estimated from Figure 5(d) (from slope and intercept, respectively). The relation for estimation of Δ*G*^*o*^ can be given as follows:

(16) $$\begin{array}{c} \Delta G^{o}=\;\Delta H^{o}-\;T\left(\Delta S^{o}\right). \end{array}$$

The estimated values of all these mentioned parameters are summed up in Table 3.

From the signs of these parameters, it has been deduced that the processes are exothermic, spontaneous, and feasible whereas from the heat of adsorption it was deduced that chemical interactions are involved in the sorption of DC [39].

### 3.7. DC Removal from Solution without and with Sorbent Pretreatments

Membranes are the porous barriers that can be effective or exclusion certain molecules that are considered as pollutants if they get entry from industry to water bodies. Each membrane has a specific exclusion limit depending on its pore's diameter and pollutant molecules' sizes. As mentioned earlier, they are installed directly in series in drainage lines and portable waters are obtained from industrially polluted waters within seconds. However, concentration polarization resulting from the accumulation of excluded molecules is a major reason for these porous compartments fouling leading to blockage of membranes, i.e., affecting membrane efficacies [13–15].

The intrinsic membrane resistance is clear from Figures 6(a), 6(c), and 6(e) (UF, NF, and RO membranes, respectively) where an initial drop in permeate flux was observed for an initial 30 min which can attribute to H^+1^ and OH^−1^ ions interaction with a membrane which is quite clear from conductance value of distilled water used (6.3 × 10^−6^ sm^−1^). The flux after time is then steady. The decline in flux in the presence of DC was more pronounced as shown in these figures which in the case of UF membrane is the adsorption of DC on the membrane which was expected to pass easily as the molecular weight cutoff of this membrane is greater than DC molecule's size. The pore size from this membrane but a concentration difference in permeate and bulk revealed its adsorption on the membrane which has blocked its pores and might be the reason for the decline in flux. In the case of NF and RO membrane, the flux decline due to DC is more pronounced as compared to UF operations as the pores in these membranes are very small, and due to concentration polarization effects, pores have been blocked.

Figures 6(b), 6(d), and 6(e) show the improvements brought about by adsorbents' pretreatment. The improvement is more pronounced in the case of MAC as after equilibration in the reactor with DC it was removed through magnet whereas there was no possibility for AC to stop its entry into membrane modules; therefore, in spite of low surface area, MAC has brought better improvements.

### 3.8. Percent Retention of DC with and without Sorbent Pretreatment

**Figure 7:**
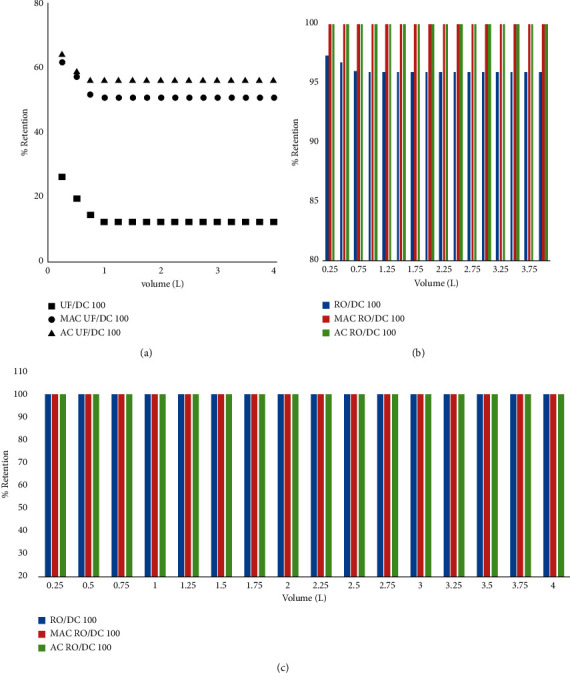
Percent retention of DC by membranes. (a) UF. (b) NF. (c) RO.

The DC percent retention without adsorbent pretreatment was from 10 to 12% which was raised and was enhanced to 51% with AC and 56% with MAC as shown in Figure 7(a). Figures 7(b) and 7(c) show a 95% and 98% retention of DC without pretreatment which was enhanced to 100% by both adsorbents. However, it should be noted that this experiment is only for comparison purposes as we are aware that the molecular weight cutoff of these two membranes is quite smaller than DC size [13–15].

## 4. Conclusions

The magnetic adsorbents are gaining popularity due to the possibility of their removal from slurry after use. In this study, an attempt has been made to control the fouling effect of DC in the case membrane system if installed for its removal in the pharmaceutical industry. The sawdust-based adsorbent AC and its counterpart MAC were prepared and characterized through different instrumental techniques. Adsorption parameters were estimated using batch adsorption approaches. The sorbents were then applied as foul controlling agents in UF, NF, and RO membranes that were caused by DC. Improved fluxes were observed with MAC due to its magnetic character. Further experiments are needed with other pollutants in this connection to validate the practicability of MAC pretreatments in membrane processes in industries.

## Figures and Tables

**Table 1:** Isothermal parameters for adsorption of DC on AC and MAC.

Isotherm models	Parameters	Adsorbents	
		AC	MAC
*Freundlich*			
	*K*_*F*_ (mg/g)	7.52	3.50
	1/*n*	0.347	0.498
	*R* ^2^	0.944	0.889

*Langmuir*			
	*q*_max_ (mg/g)	50.00	52.08
	*K*_*L*_ (L/mg)	0.05	0.02
	*R* ^2^	0.994	0.996

*Temkin*			
	*β*	12.516	11.36
	*α*	4.744	4.043
	*b*	181.345	199.799
	*R* ^2^	0.9894	0.9872

*Jovanovic*			
	*K*_*J*_ (L/g)	0.004	0.006
	*q*_max_ (mg/g)	23.15	16.20
	*R* ^2^	0.682	0.551

*Harkins–Jura*			
	*A*_*H*_ (g^2^/L)	0.43	0.28
	*B*_*H*_ (mg^2^/L)	2.35	2.25
	*R* ^2^	0.752	0.669

**Table 2:** Kinetic parameters for DC adsorption on AC and MAC.

Kinetic model	Parameters	Adsorbents and initial concentrations			
		AC 100 mg/L	MAC 100 mg/L	AC 50 mg/L	MAC 50 mg/L
*Pseudo-first-order*					
	*K*_*1*_ (1/min)	0.0361	0.0332	0.0341	0.0373
	*q*_*e*_ (mg/g)	43.39	41.07	21.96	21.82
	*R* ^2^	0.979	0.982	0.989	0.987

*Pseudo-second-order*					
	*K*_*2*_ (1/min)	9.7 × 10^−4^	6.6 × 10^−4^	2.1 × 10^−3^	1.4 × 10^−3^
	*q*_*e*_ (mg/g)	43.10	40.98	23.75	21.28
	*R* ^2^	0.994	0.983	0.996	0.987

*Power function*					
	*α*	4.14	2.32	3.45	1.01
	*b*	0.48	0.55	0.38	0.99
	*R* ^2^	0.97	0.89	0.91	1.00

*Natarajan and Khalaf*					
	*K*_*N*_ (1/min)	0.0060	0.0040	0.0070	0.0050
	*R* ^2^	0.725	0.720	0.750	0.720

*Intraparticle diffusion*					
	*K*_diff_ (mg/g min^1/2^)	2.34	2.36	1.24	1.21
	*C*	9.06	4.22	6.12	2.95
	*R* ^2^	0.816	0.822	0.806	0.807

**Table 3:** Thermodynamic parameters for DC adsorption on AC and MAC.

Adsorbent	Δ*H*^o^ (KJ/mol)	Δ*S*^o^ (KJ/mol)	Δ*G*^o^ (KJ/mol)				
			298 K	303 K	313 K	323 K	333 K
AC	−11.84	44.14	−12.92	−13.36	−13.81	−14.25	−14.69
MAC	−9.51	35.76	−10.47	−10.83	−11.18	−11.54	−11.39

## Data Availability

All available data incorporated in the MS can be obtained from the corresponding author.

## Supplementary Materials

Supplementary Materials Table S1: characteristics properties of UF, NF, and RO membranes. Figure S1: pilot plant sketch.
