# Supplementary material for: Removal of Doxycycline from Water using Dalbergia sissoo Waste Biomass Based Activated Carbon and Magnetic Oxide/Activated Bioinorganic Nanocomposite in Batch Adsorption and Adsorption/Membrane Hybrid Processes
Source: Bioinorg Chem Appl. 2022 Mar 17;2022:2694487. doi: 10.1155/2022/2694487 (PMC8947892; doi:10.1155/2022/2694487)
Supplement: Supplementary Materials — Table S1: characteristics properties of UF, NF, and RO membranes. Figure S1: pilot plant sketch. [file 2694487.f1.docx]

**Supplementary file:**

**Table S1:** Characteristics properties of UF, NF and RO membranes

| UF membrane | | NF membrane (Dow Film Tech 2.5 X 40) | | RO membrane (Dow Film Tech ECO PRO 400i) | |
| --- | --- | --- | --- | --- | --- |
| **Parameters** | **Specification** | **Parameters** | **Specification** | **Parameters** | **Specification** |
| **Membrane type** | Capillary multi bore x 7 | **model** | NF (270-2540) | **model** | RO (270-2540) |
| **Surface area** | 50 m^2^ | **Surface area** | 3.2 m^2^ | **Surface area** | 3.2 m^2^ |
| **Maximum pressure** | 109 psi | **Maximum pressure** | 100-1000 psi | **Maximum pressure** | 100-1000 psi |
| **Membrane back wash pressure** | 0.5-1 psi | **Membrane back wash pressure** | 50-800 psi | **Membrane back wash pressure** | 50-800 psi |
| **MWCO** | 100 KD | **MWCO** | 200-300 | **MWCO** | 200 |
| **Stabilized salt rejection** | 10-20% | **Stabilized salt rejection** | > 97% | **Stabilized salt rejection** | 99.5% |
| **Maximum temperature** | 40 ºC | **Maximum temperature** | 40-180 ºC | **Maximum temperature** | 40-180 ºC |
| **pH operating range** | 3-10 | **pH operating range** | 3-10 | **pH operating range** | 3-10 |
| **Back wash pH range** | 1-13 | **Back wash pH range** | 1-12 | **Back wash pH range** | 1-12 |
| **Disinfection chemicals** | Hypo chloride and Hydrogen peroxide | **Disinfection chemicals** | Hydrogen peroxide and per acetic acid | **Disinfection chemicals** | Hydrogen peroxide and per acetic acid |
| **Pore size** | 5-20 nm | **Pore size** | 5-20 nm | **Pore size** | 5-20 nm |
| **Material** | Poly ether sulfone | **Applied pressure** | 4.8 bar | **Membrane type** | Thin film composite |


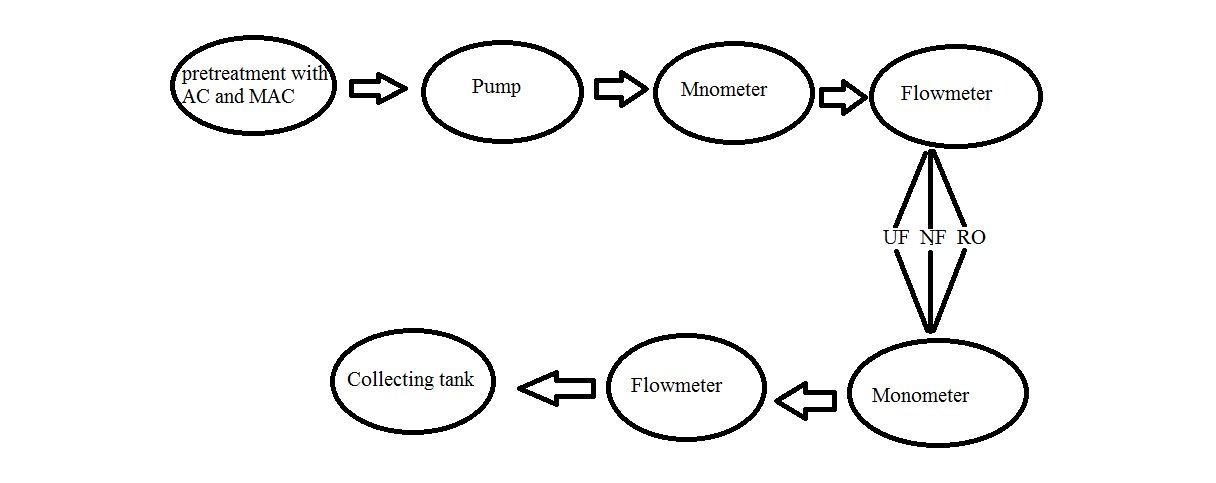


**Figure S1:** Pilot plant sketch
